# Pergola-web: a web server for the visualization and analysis of longitudinal behavioral data using repurposed genomics tools and standards

**DOI:** 10.1093/nar/gkz414

**Published:** 2019-05-20

**Authors:** Jose Espinosa-Carrasco, Toni Hermoso Pulido, Ionas Erb, Mara Dierssen, Julia Ponomarenko, Cedric Notredame

**Affiliations:** 1 Centre for Genomic Regulation (CRG), The Barcelona Institute of Science and Technology, Dr. Aiguader 88, 08003 Barcelona, Spain; 2 Institute for Research in Biomedicine (IRB Barcelona), The Barcelona Institute of Science and Technology, Baldiri Reixac, 10, 08028 Barcelona, Spain; 3 Universitat Pompeu Fabra (UPF), Barcelona, Spain; 4 Centro de Investigación Biomédica en Red de Enfermedades Raras (CIBERER), Spain

## Abstract

We present a new web application to query and visualize time-series behavioral data: the Pergola web-server. This server provides a user-friendly interface for exploring longitudinal behavioral data taking advantage of the Pergola Python library. Using the server, users can process the data applying some basic operations, such as binning or grouping, while formatting the data into existing genomic formats. Thanks to this repurposing of genomics standards, the application automatically renders an interactive data visualization based on sophisticated genome visualization tools. Our tool allows behavioral scientists to share, display and navigate complex behavioral data comprising multiple individuals and multiple data types, in a scalable and flexible manner. A download option allows for further analysis using genomic tools. The server can be a great resource for the field in a time where behavioral science is entering a data-intensive cycle thanks to high-throughput behavioral phenotyping platforms. Pergola is publicly available at http://pergola.crg.eu/.

## INTRODUCTION

The development of high-throughput platforms for the monitoring of behavior is resulting in vast recordings of time-series data (1). Often, understanding these behavioral datasets requires the simultaneous visualization of multiple data types and multiple experimental individuals (2). Although commercial recording platforms usually provide solutions to visualize and analyze this data, they are not general enough to allow the interoperability between different data types (raw, processed and environmental data for instance) or data recorded by different systems. Also, to the best of our knowledge, there are currently no software available that allow to make behavioral visualizations openly accessible or to share them with collaborators.

The main reason that hampers interoperability and shareability of longitudinal behavioral data is the lack of uniform standards (3). Interestingly, longitudinal behavioral data and genomic data share a similar structure. A sequence of behavioral events can be encoded equivalently to a sequence of genomic annotations, and the scores associated to a genomic sequence are akin to the measures associated to a behavioral trajectory or the scores derived from it. Exploiting this analogy, we developed Pergola (4), a Python library that reformats behavioral time-series data into widely-used genomic formats. The most basic functionality of this library is the mapping of time units of the former into nucleotides positions of the latter data type. In this manner, Pergola adopts formats such as the GFF (5) and the BED format (6) to represent a sequence of discrete events, such as eating bouts in feeding behavior, active states derived from posture tracking or dark and light periods of a circadian cycle. The bedGraph and the bigWig formats (7), on the other hand, are well suited to encode any type of continuous scores, examples include bout intake in feeding behavior, velocity in posture tracking or any statistical score derived from the longitudinal trajectory.

To piggyback on genomics standards has an additional advantage: there is an enormous corpus of mature software tools intended to efficiently analyze and visualize these data formats (8–10). The Pergola library consists of a set of utilities to implement ad-hoc scripting to process longitudinal behavioral data taking advantage of genomic software. The library also includes a command-line interface that wraps its main functionalities. The Pergola web-server aims to provide these utilities in a new, user-friendly interface emphasizing the intuitive visualization of the processed data. We use the Integrative Genomic Viewer (11) JavaScript plugin to enable the navigation of time series data. Such interactive navigation powers the comprehensive exploration of behavioral data at a time when novel technologies are revolutionizing the acquisition of Big Data - which is mostly longitudinal (12–14).

## WEB SERVER

### Implementation

The server backend is built on the RESTful Flask Python framework (http://flask.pocoo.org/), coupled with the Pergola library (https://github.com/cbcrg/pergola) which parses the data and is also implemented in Python. The front end is implemented in JavaScript and combines several libraries, such as Jquery (https://jquery.com) for general interaction, Bootstrap (https://getbootstrap.com) for layout definition and Handsontable (https://handsontable.com/) for spreadsheet preview and editing. The interactive data visualization is powered by igv.js (https://igv.org/), a JavaScript-based genome browser developed by the IGV team (https://github.com/igvteam/igv.js), which is embedded in the platform. Since the backend reformats the data into genomic formats, the rendering of the data works seamlessly, making it possible to take advantage of the many features offered by IGV for the interactive exploration of data.

### Data input

The Pergola server takes as input single or multiple tabulated files (CSV, TSV or XLSX) containing a series of temporal events. Files can be directly uploaded by using the ‘Input file- Choose files’ option. A typical input to the server can contain several fields among which only two are mandatory: a column containing a positive integer value designing a time point, and a column containing an associated measure. This scenario will correspond to a situation in which a variable is determined at equal time intervals (for instance velocity when tracking animal motion). Additional accepted fields include an end-of-time interval (useful when dealing with a sequence of time intervals corresponding to discrete actions such as feeding bouts), an experimental entity (to separate for instance individuals in an experiment), data types (a field that can be used to separate different types of behavioral events, e.g. eating and drinking) and an experimental phase (to distinguish between recordings acquired under different experimental conditions). All these identifiable fields constitute an ontology or a set of controlled Pergola terms. To consistently process the data, users have to assign the fields in the input file to the corresponding Pergola terms. The mapping can be set by uploading a file using the ‘Mapping file’ menu. The file should follow the GO format (http://www-legacy.geneontology.org/GO.format.ext2go.shtml) designed by the Gene Ontology Consortium community (15). Users can avoid the creation of such a file by using the ‘Design mapping’ link. This option renders the fields in the input file as a table that allows the user to directly declare the equivalence to Pergola ontology terms in a more user-friendly interface.

### Visualization options

**Figure 1.**
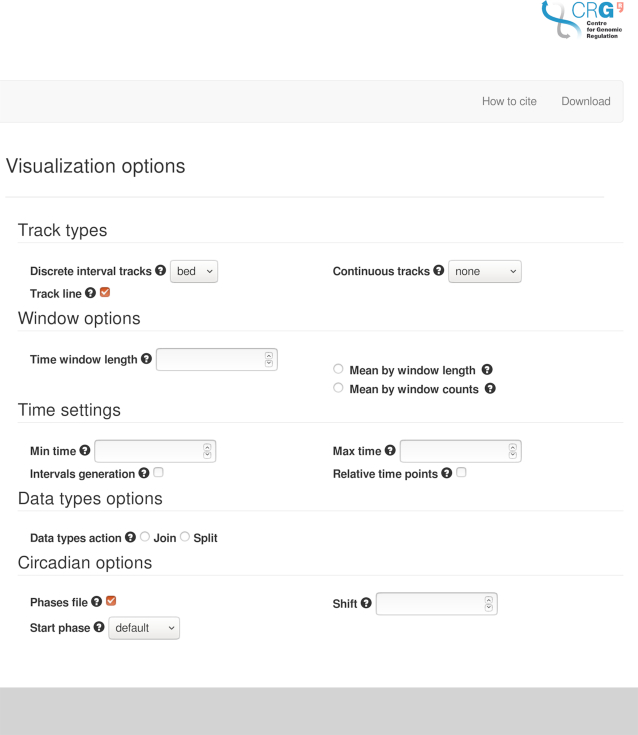
The configuration menu allows the user to choose among the main visualization/processing options of the Pergola-server.

The input file and the mapping constitute the two only requirements to submit a job. If no other options are set, by default Pergola will split the data in as many tracks as unique identifiers are found in the column tagged by the ‘track’ term in the mapping. However, the server provides various options to make the exploration of the data easier, which can be set using the ‘Visualization options’ tab on the right site of the main page (Figure 1). These options can be used both to set how the data will be rendered in the visualization and consequently, how the output data will be processed. Output data can be converted both into annotation, i.e. discrete (BED or GFF) and continuous (BedGraph, BigWig) genomic formats. Users can choose one type of format or both at the same time. Discrete tracks are displayed as fixed-height blocks, and the blocks (events) assigned to different ‘data_types’ (Pergola term to map different type of annotations on the same track) are colored differently for easier recognition. On the other hand, continuous tracks allow numerical values tagged by the ‘data_value’ term to be depicted as vertical bars in the corresponding track, where height is proportional to value. This allows to easily perceive differences between tracks at a given point provided all the tracks are rendered using the same data range. Some third-party genome browsers use the so-called track line to describe some of the graphical parameters that have to be used when a BED file is displayed. We allow the user to decide whether or not to include it, since some genomic analysis tools do not work when the file includes this line.

When choosing to format the output into continuous tracks, the data can be additionally binned into windows of time. This option becomes active when the user sets a window size introducing a value in the ‘Time window length’ option. By default, the data values restricted to a given time window will be summed up and shown in the corresponding track as bars of the resulting height expanding the window size. Alternatively, instead of summing up data values within the window interval, they can be averaged over either window length or over all time points within the window by using the ‘Mean by window length’ and ‘Mean by window counts’ options, respectively. This kind of binning of the data helps users to visually compare quantitative tracks.

Users can also choose to restrict the exploration of the input data to a defined time interval by setting the minimum and/or the maximum time point to be processed using the ‘Time settings’ menu (‘min time’ and ‘max time’ options, respectively). Moreover, when the ‘Relative time points’ option is selected, absolute time points are referenced to the initial time point of the data (i.e. the first time point becomes zero), and displayed as such in the embedded genome browser. This operation is useful when data has been recorded using real timestamps. Finally, the ‘Intervals generation’ option is also found under this menu. This option is required when the input data only contains a single time point for each recorded event, a situation that prevents its visualization in the browser. In these cases, Pergola creates intervals that correspond to *t_n_, t*_*n*+1_ – 1 and assigns to the interval the value from the point *t_n_* (where *t_n_* specifies a given time point and *t*_*n*+1_ is the time point after, *t*_*n*_).

Additionally, if the user has tagged a column as ‘data_types’ in the mapping, it is possible to decide whether to separate the data by these data types in different tracks or to maintain them in a single track. If an input file contains both recordings of feeding and drinking events, e.g., if the user chooses the ‘split’ option, the server will generate separate tracks for feeding and drinking for each individual.

**Figure 2.**
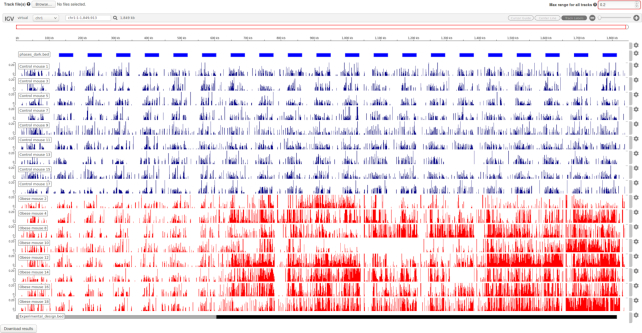
Visualization of our sample data. This dataset corresponds to a collection of 17 mice feeding behavioral recordings restricted to 3 weeks of the original study (17). During a one-week period, all the animals received a normal chow to establish the basal behavior. After this period, mice were divided into two groups, a control group that continued under a normal chow regime and an obese group that was exclusively offered a hypercaloric diet. The visualization of this data can be very helpful to identify differential patterns unfolding along time. In this screenshot, the top track (blue) displays the night periods (mice are nocturnal animals), the red (control mice) and blue (obese mice) tracks represent the food consumed during a five-minutes time window by an individual mouse. Finally, the bottom track depicts a period within which all the animals eat normal chow (gray), followed by a period where obese animals received only a hypercaloric diet. It is clear that upon introduction of the hypercaloric food the feeding behavior of the obese group is disrupted and loses its circadian rhythmicity.

Some behaviors are heavily influenced by environmental signals. Circadian rhythms constitute a prime example where a behavior is synchronized to the presence or absence of an environmental cue (i.e. presence or absence of light along the day) (16). In this case, researchers may want to explore whether a behavioral recording remains coupled after a given experimental intervention. Our server offers some utilities to facilitate this type of data exploration. Users can choose to generate BED tracks depicting day and night periods, which can help in the identification of circadian coupling or disruption patterns along the data tracks within these phases in the final visualization, as shown in Figure 2. These options can be found under the ‘Circadian options’ menu.

### Interactive visualization

We placed special emphasis on data navigation, since behavioral longitudinal data often presents visual patterns that enhance its understanding. Thanks to the Pergola server, behavioral scientists can now interactively display their data and explore it. Data visualization is automatically rendered once the data is processed according to the options set by the user. The visualization is powered by the IGV.js plugin, which provides a flexible and user-friendly navigation interface allowing to zoom into given periods of interest. Each individual track can also be featured to display a given data range or color to display tracks belonging to individuals of the same experimental condition. The results tab also allows to upload additional files for visualization using the ‘Track file’ button (Experimental design track on Figure 2). These files can display relevant information that was processed in previous executions or produced by the Pergola command-line interface.

### Data output

Besides presenting the data, the server allows to download the genomic format tracks for further processing. The resulting files, together with the input files, can be freely downloaded as a Zip archive using the ‘Download results’ button. This feature allows users to use their preferred desktop genome browser for the visualization of the results or—as described in detail in the data sharing point below—to upload the results again to the server at any time. Perhaps the most interesting option, as described in our previous work (4), is that the user can now perform additional analyses using the plethora of tools supporting the genomic formats involved.

### Data sharing

Data sharing acquires a paramount importance in behavior-related disciplines, since often experiments involve the collaboration of multidisciplinary teams and its results have to be explored by end users with limited bioinformatics skills. For this, results from any submission on our server are available up to 10 days on the host server and can be accessed and shared with other collaborators using the browser unique URL resulting from the submission. To enable the sharing of results during longer time periods, the results saved from the server using the download button (provided in a single packaged Zip Archive) can be rendered again by using the ‘Upload data’ link on the server home page, either by submitting the entire Zip archive or all the files separately (track, chromosome files, etc.). Note that this feature can be used to upload and inspect any output files resulting from Pergola command-line applications as if they had been originally processed starting from the web interface.

## CONCLUSION

In this article, we introduce a new web application to process and visualize time-series behavioral data, the Pergola web server. Due to the increasing interest in the analysis of behavioral time series data (18), there is a need for tools that enable the comprehensive analysis of complex experiments involving multiple individuals and multiple data types (19). Our web application aims to provide a user-friendly web interface with a strong focus in data exploration to allow the user to share and analyze their data. At a time when novel technologies enable the acquisition of large amounts of data for the understanding of behavior (20,21), Pergola can become a useful resource for the behavioral community. Finally, we think the server can be a valuable resource for researchers working with other types of longitudinal data.
